# Retention and Suppression of Urine

**Published:** 1875-08

**Authors:** R. J. Preston

**Affiliations:** Virginia


### RETENTION AND SUPPRESSION OF URINE.

BY R. J. PRESTON, M.D., OF VIRGINIA.

*Read before the Abingdon Academy of Medicine, July 5th, 1875.*

The subject for discussion to-night, gentlemen, is one of very great importance to our profession, as it is these conditions occasionally met with in the practice of every physician, which demand at his hands prompt and energetic measures to ward off impending danger, as upon the speedy success of these efforts and measures the life of his patient often directly depends; and it is these conditions too, alarming as they sometimes are, and speedily threatening a fatal termination, that often give the physician or surgeon most trouble, require the exercise of his best skill, and exhaust, to the utmost limit all his therapeutic and surgical resources for their relief. Associated, as they are, with a variety of diseases, and symptametic of different conditions of the urinary or general system, the thorough and complete study and discussion of "Retention and Suppression of Urine" would lead us into a wider field than our time and space will permit. We will, therefore, for the present endeavor merely to enumerate some of the causes of these conditions, and some of the diseases in the course of which they must occasionally be met with by every practitioner; and then proceed to discuss some of the various methods of treatment, and the difficult measures used for their relief, as have seemed to us most beneficial and successful.

*Retention of urine* may be caused by mechanical obstruction, as calculus in the urethra or in the bladder; by stricture, organic or spasmodic, of the muscular fibres of the urethra, or spasmodic contraction of the sphincter-vesical muscle, excited by the action of certain remedies, as cantharides, turpentine, etc., or by cystitis from any cause; by an enlarged prostate, or by paralysis of the bladder, as in typhus or typhoid fevers, and other low and debilitated states of the system.

*Suppression of urine* usually occurs in advanced age, but is not necessarily limited to any period of life. The usual causes are acute congestion, or inflammation of the kidneys, organic renal disease, obstructed ureters, from nephritic calculi and other causes, and low debilitated states of the system. Exposure to cold, large doses of cantharides, turpentine, etc.; acute inflammatory diseases, as the exanthemata, etc., may produce it; and it may occur from heart disease, or from pulmonary obstruction, or from an obstructed renal circulation by pressure, as in pregnancy, or from abdominal tumors.

In all serious diseases the practitioner should never forget to ascertain whether his patient is regularly secreting and voiding his urine; and where such is not the case, then the differential diagnosis between these two conditions is important. Palpation and percussion will generally be sufficient to determine the presence or absence of urine in the bladder, but sometimes the use of the catheter will be required. By these means, and sometimes by the presence of pains and tenderness over the loins, the differential diagnosis may be made, and the cause in obscure cases referred back to the kidneys. Under all circumstances, suppression of urine from inaction of the kidneys is an alarming symptom, and, if long continued, proves fatal by uræmic poisoning.

In the treatment of these conditions the important and leading indication always is to ascertain and remove the cause. This will doubtless, in the great majority of cases, be easily and safely effected; but we all are apt to meet with cases occasionally which defy all our skill, and refuse to yield to any and every measure brought to their relief. And in many chronic and obstinate cases of long standing presenting these symptoms, and demanding immediate relief, the entire urinary apparatus is so completely deranged and disorganized as to obscure, in a great measure, our explorations and diagnosis, and render uncertain, and sometimes dangerous, the ordinary methods of relief. In cases of retention of urine from calculi, stricture, etc., hot baths, opiates, anæsthetics, etc., will generally suffice for relief, or the use of the catheter may be required. Opium and belladonna by enemata or by suppositories, or morphiæ hypodermically, are effective remedies. Operative procedures must be resorted to in cases of organic stricture, vesical calculi, etc., whenever the condition and urgency of the case demand it. Where the retention is due to paralysis of the bladder, as in low fevers, etc., the catheter must be regularly employed once or twice daily.

The prostatic enlargements of old age are looked upon as almost an opprobrium of the healing art, and generally bring about a concomitant train of symptoms, connected with the urinary organs, of which these we are considering are not the least annoying and troublesome.

In these cases, in addition to the use of the above-mentioned remedies for the relief of acute symptoms, measures should, I think during the intervals, be directed to the removal or reducing of these enlargements, as far as may be possible; and to this end, besides other remedies, I think that I have used with most success iodoform in suppositories, with belladonna, etc., once or twice daily.

When hæmorrhoids are associated with these enlargements of the prostrate gland, which is often, if not generally, the case, (and I have seen hæmorrhoids alone produce retention of urine,) they should be removed as early as possible, by operative procedures when practicable.

And in this connection I desire to call attention to a mode of catheterism in these difficult cases, which I have used many times, and have been led to look upon as safer and easier than most other methods. It is to take an ordinary gum catheter—smallest size if necessary—with the wire in it, and bent in the shape of a silver catheter or sound; introduce it into the urethra as far as possible, up to the obstruction; then withdraw the wire a little at a time while gently pressing forward; the end of the gum catheter being thus made flexible will generally override the obstructions and pass through the sinuosities of the prostate gland when no other instrument will. I have used this method successfully in some very difficult cases, and have seen the impression of these irregular sinuosities of an enlarged prostate plainly marked upon the end of the catheter after it had been retained for a short while.

In cases of suppression of urine, if partial and transiently occurring, as in measles, scarlatina, etc., diuretics, together with hot baths and counter-irritation over the lumbar region, may suffice to relieve it; but in the graver cases connected with inflammatory and organic diseases of the kidneys, the treatment is not as easy, nor success so sure. This accidental symptom, which may arise at any time in these diseases, or in other severe constitutional diseases, as above mentioned, is always alarming, and must be met by prompt and negative measures for its relief, aside from the regular treatment of the disease in the course of which it is met.

Cupping, or blistering the loins, purgatives, diaphoretics and sudorifics as hot baths, steam baths, etc., are indicated in proportion to the threatening condition of the patient, and the danger of uræmic poisoning. Active diuretics here are generally hazardous and contra-indicated, and our efforts should be directed mainly to bringing about vicarious or supplemental discharges through the bowels, skin, etc., and thus relieve the kidneys, eliminate the urea, and ward off the impending danger.

Where we can excite the functional activity of the skin, and bring about profuse diaphoresis, we are doing much to fulfill these indications; and while sustaining and building up the system by the use of tonics and nutritious diet, we can often excite and gradually restore the functional activity of the kidneys by the use of the mildest diuretics, counter-irritation over the loins, etc., and thus accomplish a perfect restoration to health in some of the worst cases. But to discuss all the treatment necessary to these conditions in different cases would lead us, as above remarked, into the discussion of several different diseases; so we will close, hoping that these few desultory thoughts may not prove entirely unprofitable, but may serve at least to call forth more that will be said to advantage by other older and more experienced members of this Academy in the discussion of this question.

